# A Review of the Nonpressor and Nonantidiuretic Actions of the Hormone Vasopressin

**DOI:** 10.3389/fmed.2015.00019

**Published:** 2015-03-24

**Authors:** Gaurang P. Mavani, Maria V. DeVita, Michael F. Michelis

**Affiliations:** ^1^Division of Nephrology, Department of Medicine, Lenox Hill Hospital, New York, NY, USA

**Keywords:** arginine vasopressin, metabolic syndrome, social behavior, osteopenia, cell proliferation, hypertension, chronic kidney disease, inflammation

## Abstract

The pressor and antidiuretic actions of arginine vasopressin (AVP) have been well documented. This review focuses on the less widely appreciated actions of AVP which also have important physiologic functions and when better understood may provide important insights into common disease states. These actions include effects on pain perception and bone structure as well as important relationships to the varied components of metabolic syndrome. These include effects on blood glucose, lipid levels, and blood pressure. AVP may also play a role in the progression of chronic kidney disease and effect physiologic changes relating to aging, abnormal social behavior, and cognitive function. Important cellular responses including cell proliferation, inflammation, and control of infection and their relationship to AVP are described. Finally, the effects of AVP on hemostasis and the hypothalamic–pituitary–adrenal axis are noted. The goal of this summary of the various actions of AVP is to direct attention to the potential benefits of research in these underemphasized areas of importance.

## Introduction

Arginine vasopressin (AVP), also known as antidiuretic hormone, is a small peptide with only nine amino acids. It closely resembles oxytocin differing from it by only two amino acids.

The main site of AVP production is the hypothalamus. Smaller amounts are also produced outside the hypothalamus in many tissues locally. Thus, apart from its endocrine effect, AVP also exerts autocrine and paracrine effects. The tracts from the hypothalamus containing AVP terminate in the posterior pituitary, also called the neurohypophysis, where AVP is stored. These tracts also innervate other areas of brain and the spinal cord enabling AVP to exert its actions not only systemically but locally in the brain and spinal cord. The most sensitive stimulus for the secretion of vasopressin is serum osmolality. Although the secretion of AVP is also affected by changes in blood volume and blood pressure (BP), these responses require larger variations than changes in serum osmolality (1).

Vasopressin, being a small peptide, is easily filtered through the glomerulus. It is not metabolized in the kidney being excreted unchanged in the urine. Under normal physiological conditions, the concentration of AVP in the plasma is very low (<2 pg/mL) making it difficult to measure. Copeptin is a surrogate marker of vasopressin secretion. Copeptin is a peptide which is secreted along with AVP in equimolar amounts and is easier to monitor as its half-life is longer and it is more stable (2).

Arginine vasopressin acts primarily through its receptors which are located in the brain as well as in the periphery. These receptors are named V1a, V1b, and V2 (Table 1). V1a receptors are present in many tissues including the smooth muscle cells of vessels, as well as the brain, adrenal cortex, adipose tissue, and hepatocytes. V1b receptors are mainly present in anterior pituitary, adrenal medulla, islet cells of Langerhans, and white adipose tissue. V2 receptors are present in the kidney on the basolateral membrane of the collecting duct and alveolar epithelial cells (3).

**Table 1:** **Arginine vasopressin receptors and their locations**.

V1a receptors	V1b receptors	V2 receptors
Smooth muscle cells	Anterior pituitary	Basolateral membrane of collecting ducts
Brain	Adrenal medulla	Alveolar epithelial cells
Adrenal cortex	Islet cells of Langerhans	Osteoblasts
Adipose tissue	White adipose tissue	Osteoclasts
Hepatocytes		
Osteoblasts		
Osteoclasts		

**Figure 1:**
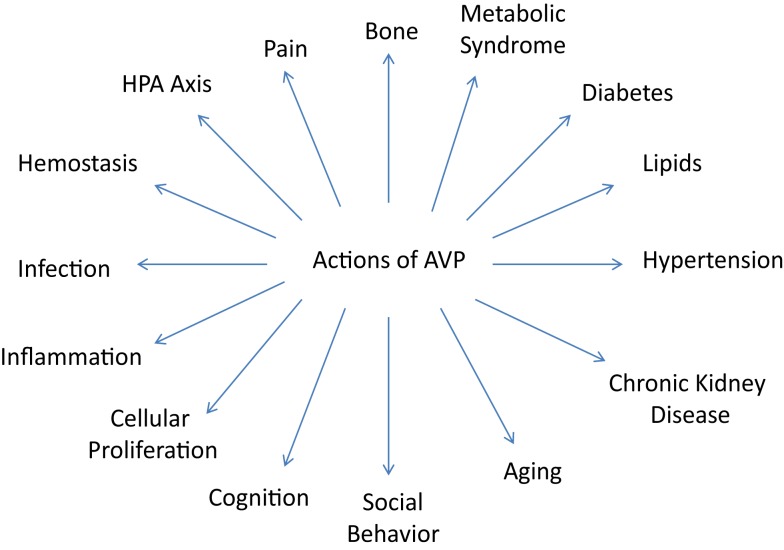
**The varied nonpressor and nonantidiuretic actions of vasopressin are illustrated**.

In addition to the well known effects on BP and the kidney, AVP has also been demonstrated to have numerous other significant actions centrally and in the periphery due to its effects on the various V1a and V1b receptors. It is these varied and important actions that will be the focus of this comprehensive review and which are illustrated on Figure 1.

## AVP and Pain

Arginine vasopressin has a significant effect on pain perception via the central nervous system through receptors V1a and V1b located on neuronal cells including those distributed throughout the hypothalamus. In the brain, these neurons extend to the lateral septum, hippocampus, amygdala, and other brain structures. Studies have shown that AVP can increase the pain threshold to painful stimuli (4).

It has been demonstrated that in response to stress and pain, central as well as peripheral levels of AVP are increased. It is thought, however, that the analgesic actions of AVP are not mediated by peripheral AVP as the blood–brain barrier (BBB) prevents the passage of AVP from the periphery to the brain. For example, peripheral injection of AVP in rats did not reduce the pain threshold when rats were given pain in a graded manner (4). In contrast, further experiments showed that pain perception can be altered by AVP. Pain was induced in a graded manner by using techniques like hot plate and tail flick methods. The initial threshold at which pain occurred was noted. AVP was then given by an intraventricular route and the pain stimulus was reapplied. The pain threshold was now significantly higher (4). In another experiment, a pain stimulus was applied to Brattleboro rats which are deficient in vasopressin. These rats showed an exaggerated pain response. When AVP was given intraventricularly, the pain threshold increased indicating that the analgesic deficit was AVP-dependent. Furthermore, the intracerebral action of AVP was blocked by injecting antiserum against AVP into the ventricle resulting in a decreased threshold to painful stimuli (5).

In another study, Ahn et al. showed in his animal experiments that the analgesic actions of AVP were blocked by a V1a and not a V2 receptor antagonist, suggesting that AVP exerts its antinociceptive activity exclusively through V1a receptors (6). Watkins et al. showed that not only intracranial but intrathecal injections of AVP can cause an antinociceptive effect which suggests that the antinociception action of AVP extends to the spinal cord (7). Yang et al. demonstrated that, in acute headaches, intranasal AVP was effective in reducing pain. Intranasal AVP can be effective since there is a pathway connecting the olfactory neuroepithelium and brain which enables AVP to bypass the BBB (8). In summary, multiple studies have confirmed the analgesic actions of AVP.

## AVP and Bone

Pituitary hormones including AVP have a significant effect on bone. Direct control is exerted through vasopressin and oxytocin. Indirect control is exerted by thyroxine, gonadal hormones, and adrenal hormones. These latter hormones are in turn controlled by pituitary hormones. Oxytocin exerts significant osteoblastic activity by acting directly on the bone. Mutation of the oxytocin receptor on osteoblasts can cause significant osteopenia. Oxytocin and AVP are also produced locally in tissues and it is likely that locally produced pituitary hormones may take part in skeletal stability. AVP is a key regulator of bone remodeling by controlling both osteoblast and osteoclast activity through its receptors. Osteoblasts and osteoclasts both have AVP receptors V1a and V2, and their activation can cause stimulation of kinases. The latter have been shown to favor stimulation of osteoclasts causing bone resorption and osteoporosis while inhibiting bone formation by osteoblasts. Conversely, antagonists to either V1a or V2 receptors favor osteoblasts and new bone synthesis while inhibiting osteoclasts by mechanisms as yet not completely understood (9).

Furthermore, approximately 50% of chronic hyponatremic syndromes result from the syndrome of inappropriate antidiuretic hormone secretion (SIADH). One-third of body sodium is stored in the bones. Thus, in chronic hyponatremia due to SIADH with excess AVP activity, there may be bone resorption by osteoclasts and also by the release of sodium from the bone (10).

In addition, experiments by Barsony et al. demonstrated that hyponatremia causes direct stimulation of osteoclastogenesis and osteoclast resorption in young rats. Experiments revealed that low sodium in the medium directly stimulated osteoclast progenitor cells to produce osteoclasts. They also found that this effect was not due to hypoosmolality but was due specifically to the hyponatremia. They exposed the murine preosteoclastic cells to low sodium in the medium but maintained the osmolality at normal levels by adding mannitol. In this situation, the conversion of preosteoclastic cells to osteoclasts persisted. In fact, it was shown that hypoosmolality was protective of bone as low osmolality induced stretching of cells and increased intracellular calcium which causes apoptosis of the osteoclasts. Thus, the hyponatremia was responsible for the increased osteoclastogenesis and increased osteoclast activation. Increased osteoclastogenesis was seen even with mild hyponatremia (11).

It also appears that hyponatremia can enhance osteoclast proliferation by inducing oxidative stress. Vitamin C is considered to be a strong defense against reactive oxygen species. Entry of vitamin C into the cell is sodium-dependent and is mediated by a sodium-dependent ascorbic acid transporter. This transporter is modulated by extracellular sodium and it has been shown that even a small reduction in extracellular sodium causes a marked decline of the uptake of radiolabeled vitamin C. At the same time, it was shown that the accumulation of reactive oxygen radicals was increased as a result of reduced intracellular vitamin C and was directly proportional to the level of hyponatremia. Also, evidence of oxidative DNA damage was found (11).

Other studies have described an association between endurance exercise and athlete-related osteopenia. Endurance athletes are prone to exercise-associated hyponatremia in part related to the high rate of hypotonic fluid intake during and after exercise. Persistent secretion of AVP contributes to the hyponatremia. Since bone is thought to buffer significant changes in plasma sodium, Butler et al. studied this association in endurance athletes. Results showed that endurance athletes had both pre- and post-race plasma sodium levels in the normal range although post-exercise sodium was lower than pre-exercise sodium. The change in sodium level pre- and post-exercise was directly related to the change in bone mineral content. AVP could be related to osteopenia directly through its action on osteoclasts and indirectly by hyponatremia causing the release of sodium from bone (12).

Finally, it has been observed that patients with chronic hyponatremia have an increased incidence of osteoporosis and there is an association of hyponatremia with falls and fractures in elderly patients. A recent study revealed that 9.1% of patients seen in an emergency setting for fracture were hyponatremic (13).

## AVP and Metabolic Syndrome

The metabolic syndrome (MetS) is a complex disorder and is defined by interconnected factors which include hypertension, obesity, insulin resistance, pro-inflammatory and pro-thrombotic state, dyslipidemia, including elevated triglycerides (TGs) and apolipoprotein B-containing lipoproteins, and low high-density lipoproteins, non-alcoholic fatty liver disease, and sleep apnea. MetS increases the risk of diabetes mellitus (DM) and coronary artery disease (CAD). Traditional risk factors for MetS are well known but it is still debated if this is a distinct syndrome or a marker of multiple risk factors which places an individual at high risk for DM and CAD. Biomarkers of MetS are insulin resistance, leptin, and changes in epinephrine and norepinephrine, oxidized low-density lipoprotein cholesterol, uric acid, C-reactive protein (CRP), plasminogen activator inhibitor-1, and aldosterone (14). The pathophysiology of MetS is poorly understood. It is observed that even with similar risk factors, age of onset of MetS varies. This suggests that genetic and environmental factors play an important role. It is also increasingly recognized that psychosocial stress and dysregulation of hypothalamo–pituitary axis and autonomic nervous system may play a vital role (15).

Arginine vasopressin has diverse actions which include vasoconstriction, platelet aggregation, stimulation of glycogenolysis from liver, effects on lipids, regulation of ACTH secretion from the pituitary, and release of insulin and glucagon from the pancreas. Thus, AVP may play a role in the development of MetS (14). It has been postulated that psychosocial stress may be an etiologic factor in MetS. Normally, corticotropin-releasing hormone (CRH) and AVP act on the pituitary to release ACTH. However, psychosocial stress amplifies the action of AVP on the pituitary through V1b receptors to release ACTH. This increase in ACTH causes increased stimulation of the adrenal cortex and results in high serum cortisol levels. Mice that lack V1b receptors have a lower level of serum cortisol at stress and at basal state. High serum cortisol causes obesity, hyperglycemia, and insulin resistance. Moreover, ACTH released due to AVP stimulation does not respond to negative feedback by serum cortisol in contrast to CRH-induced ACTH release which does respond to cortisol levels. AVP has also been shown to directly simulate the synthesis and release of cortisol through V1a receptors in the adrenal cortex (15, 16).

It is difficult to measure AVP as its half-life is only 24 min and it is rapidly cleared from plasma and also it is attached to platelets. Copeptin is a surrogate marker of AVP and its level correlates to levels of AVP in plasma. It is easy to measure copeptin as it is quite stable, has a long half-life, and is not attached to platelets. Saleem et al. reported elevated copeptin levels in association with MetS. Moreover, those who had the highest copeptin levels, in the third or fourth quartiles for plasma copeptin, had 70–100% higher odds of MetS compared with those in the bottom quartile. Individuals with the highest number of MetS components had higher copeptin levels. Plasma copeptin was also associated with higher TG levels and lower HDL cholesterol levels and inversely associated with physical activity (14).

In a study done by Enhorning et al. in Sweden between 1991 and 1994, 2064 patients were serially followed for 15.8 years. Baseline copeptin levels were measured. At the end of 15.8 years, an oral glucose tolerance test and measurement of MetS individual components were done in these subjects. They found that copeptin levels at baseline could predict the development of abdominal obesity and incident DM. Baseline copeptin levels could also predict the future incidence of diabetes even without impaired fasting blood glucose. Thus, copeptin could be used as a screening tool to detect high-risk individuals who could develop DM in the future even though they had a normal baseline fasting glucose levels. They also found that the association between baseline copeptin and diabetes was independent of the development of obesity. Further, copeptin levels at baseline were associated with microalbuminuria after long-term follow-up and this was independent of baseline MetS variables. This finding may be mediated by the action of AVP on V2 receptors. In the rat model, chronic infusion of desmopressin (DDAVP) caused an increase in proteinuria and AVP suppression caused a decrease in proteinuria and improvement in renal function (16).

## AVP and Diabetes

Vasopressin receptors are present in the liver and pancreas. V1a receptors are found in hepatocytes and V1b receptors are found in the cells of the islets of Langerhans in the pancreas. In liver, AVP causes glycolysis thus elevating plasma glucose. Glucagon also produces the same effect on the liver although it does not act through V1a receptor. AVP can still produce this effect even when the glucagon receptors are blocked since the receptors of AVP and glucagon in the liver are different. V1b receptors are present in both alpha and beta cells of the islets of Langerhans. Thus, stimulation of islet cells causes stimulation of both insulin and glucagon depending on extracellular glucose concentration. Infusion of AVP causes a significant increase in plasma glucose level in subjects with normal serum glucose. The effect of AVP on glucose metabolism was demonstrated in animal experiments and human trials. In an experiment with mice, the genes for V1a and V1b receptors were knocked out. These mice had altered glucose homeostasis and increased catabolism of fat suggesting that AVP may play a role in metabolic disorders and glucose metabolism (3).

In addition, in human studies, it was found that people who have a variant of *AVPR1A* (which encodes the V1a receptor), had a higher fasting blood glucose level and an increased prevalence of diabetes (17). Roussel et al. showed an inverse association between water intake and the risk of hyperglycemia during a 9-year follow-up. He compared the risk of developing diabetes in the future based on current water intake. He noted that people who drank more than 1 L of water had 27% less chance of getting diabetes than people who drink less than 0.5 L/day. This observation was made after adjustment for confounding variables. This study proposes that people who drink less water have a greater chance of developing diabetes related to higher AVP levels (18).

Patients with Type I diabetes have selective depletion of beta cells. They have impaired secretion of insulin but have intact secretion of glucagon (alpha cells). Not only are the actions of insulin and glucagon antagonistic, but secretion of insulin causes inhibition of the secretion of the glucagon. Therefore, in patients with type 1 diabetes, secretion of glucagon is significantly increased as there is no inhibition by insulin thus elevating the blood glucose level. Therefore, in Type 1 diabetes, even physiological levels of AVP can cause a profound increase in blood glucose by stimulating glucagon secretion resulting in increased production of glucose by the liver (19).

To prove an association between AVP and glucose, Hsu et al. conducted experiments with rats that were diabetic and non-diabetic. Diabetes in rats was induced by the infusion of streptozotocin. He initially measured basal secretion of glucagon in both groups of rats before AVP was infused. He then infused AVP and noted that the secretion of glucagon was double in the diabetic rats compared to non-diabetic rats. Thus, the diabetic rat pancreas was more sensitive to AVP in respect to glucagon secretion. They also found that at baseline, before AVP was infused, diabetic rats had a higher concentration of AVP in plasma than non-diabetic rats. This study also showed that a lower concentration of AVP is needed to increase glucagon secretion but a higher concentration of AVP was needed to secrete insulin. This suggests that alpha cells are more sensitive to AVP than beta cells. AVP thus exerts greater influence on glucagon secretion than insulin secretion at basal levels. Since V1b receptors are involved in the stimulation of glucagon, this study highlights that in the future, a V1b receptor antagonist may play a role in the treatment of diabetes by inhibiting unopposed glucagon secretion (19).

Also noteworthy, Dheen et al. has shown that in diabetic rats, there is hypertrophy of the supraoptic and paraventricular nuclei which suggests that there may be hyperactivity of neurons that secrete AVP in diabetic rats (20). It has also been shown that the concentration of AVP in the pancreas of humans and rats is much higher than the concentration present in the serum. AVP is found in the perivascular compartments of the pancreas and not in islet or acinar cells. Thus, it is likely that locally formed pancreatic AVP rather than pituitary AVP may play an important role in glucagon production (3, 19).

Finally, Pasquali et al. showed that in obese male subjects when CRH and AVP were infused, there was an exaggerated pituitary response with an increased release of ACTH and a higher cortisol level resulting in hyperglycemia. Thus, AVP has a role in causing hyperglycemia by not only acting on islet cells but through its action on V1b receptors in the pituitary (15). The various cells and receptors that influence blood glucose levels are summarized in Table 2.

**Table 2:** **Cells, receptors, and the effects of arginine vasopressin on blood glucose levels**.

Hepatocytes V1a – glycolysis
Beta islet cells V1b – insulin release
Alpha islet cells V1b – glucagon release
CNS (pituitary) cells V1b – ACTH release increases glucocorticoids
Adrenal cortex V1a – increases glucocorticoids

## AVP and Lipids

Arginine vasopressin has been shown to control fat metabolism by multiple mechanisms. Centrally, it stimulates the sympathetic nervous system which in turn affects lipid metabolism (21). Peripherally, AVP regulates lipid metabolism by regulation of several hormones affecting fat metabolism such as insulin, glucagon, glucocorticoids, and epinephrine. AVP also inhibits tissue lipase by its direct action on AVP receptors. AVP also affects the glucose level, the substrate needed for fat metabolism (3).

Injection of AVP in the lateral ventricle of rat causes stimulation of the sympathetic nervous system by a mechanism not well understood. Sympathetic nervous system stimulation causes the release of epinephrine which in turn causes stimulation of the metabolism of TGs in adipose tissue. Peripheral nerve terminals supply adipose tissue as well as the blood vessels of adipose tissue. It has been shown by various studies that any interruption to these nerve terminals to adipose tissue will affect lipid metabolism. In human studies, infusion of epinephrine and norepinephrine lead to elevation of free fatty acids (FFA) which are a byproduct of TG metabolism (22).

Hiroyama et al. has shown in rat experiments that AVP has an antilipolytic effect involving V1a receptors. AVP exerts antilipolytic action by inhibiting tissue lipase thus preventing the breakdown of TG. In his experiment, he infused AVP in V1a-deficient rats and he noted that these rats had increased lipolysis and increased serum levels of glycerol and ketone bodies. AVP is also known to inhibit the actions of isoproterenol. In V1a-deficient mice, isoproterenol was infused and it was noted that in brown adipose tissue (BAT), lipolysis was increased threefold (22). Stimulation of lipid metabolism in BAT is known to increase core temperature. Okuno et al. in his experiments with rats has shown that AVP infusion caused a decrease in the core body temperature due to inhibition of lipid metabolism in the BAT. He noted that even in rats with an anterior hypothalamic lesion, the infusion of AVP reduced core body temperature. Thus, he concluded that this effect of AVP was not centrally mediated but was peripheral due to the inhibition of fat metabolism (23).

Insulin is the most potent antilipolytic hormone. Thus, insulin therapy for diabetes causes obesity by inhibiting fat metabolism. Insulin mediates this action by reducing the level of cyclic AMP in cells. Insulin signaling is done through kinase Akt and p70S6 kinase. These kinases are activated by AVP such that AVP not only causes stimulation of insulin release from islet of Langerhans but also enhances insulin signaling. In V1a-deficient rats, insulin signaling is impaired which in turn causes insulin resistance. Thus, V1a-deficient rats demonstrate increased lipolysis secondary to insulin resistance. AVP can control lipid metabolism by controlling the glucose needed for lipid metabolism. AVP stimulates glycogenolysis in the liver by stimulating glucagon and epinephrine which in turn cause an increase in glucose levels. Glucose is metabolized in the adipose tissue to TG. Insulin facilitates this conversion (22).

V1b receptors are present on the beta cells of the islets of Langerhans. Fujiwara et al. in his experiments with V1b-deficient mice showed that the serum level of insulin was reduced. This was due to reduction of insulin release from the islets of Langerhans. He also noted that these mice had increased insulin sensitivity which probably acts as a compensatory response to low serum insulin levels. Also, in these mice, lipolysis was impaired since V1b receptor deficiency may stimulate lipogenesis by increasing insulin sensitivity (24).

## AVP and Hypertension

The interplay of the sympathetic nervous system, the renin angiotensin aldosterone system (RAAS), and AVP relate to systemic BP. It has been shown that on a day-to-day basis, AVP does not play a significant role in the maintenance of BP (25). Landry et al. in his human experiments, however, demonstrated that vasopressin deficiency contributes to the vasodilatation of septic shock (26).

Black people have a higher incidence of hypertension than white people. The level of AVP has also been shown to be higher in black individuals. Urine volume was lower in blacks compared to whites and urine concentration was higher in black people compared to whites especially in the daytime. Also, studies found that even when BP was in the normal range, pulse pressure was higher in black individuals. There was a strong statistical association between higher urine concentration and high pulse pressure in black men. Black men and women were compared with each other regarding urine volume and urine concentrating ability and it was shown that men had a higher ability to retain salt and concentrate urine than women. Increased conservation of sodium caused less nocturnal dipping of BP in black men and increased conservation of sodium in day time resulted in a higher BP at night (27). Choukroun et al. showed that healthy subjects who drank smaller amounts of water excreted sodium more slowly compared to healthy subjects who drank a larger amount of water (28). Bankir et al. made an association between urine flow rate and urine sodium excretion in healthy subjects. The results showed that urine sodium excretion was independent of urine flow rate as long as urine flow was higher than 1 L but once urine flow was less than 1 L, sodium excretion decreased. This suggests that a higher level of vasopressin is required for sodium conservation and sodium is absorbed only when the AVP level reaches a certain threshold. A higher vasopressin level in black people compared to whites could be due to a change in the set point of the hypothalamic osmostat to lower levels of serum osmolality. This higher AVP could be a survival advantage in black people for facilitating water conservation in adverse drought and famine conditions. This adaptation could cause decreased sodium excretion. In black people, RAAS activity is low. This may explain why ACE inhibitors are not as effective in black hypertensive patients (27).

It has also been shown in humans that acute stimulation of V2 receptors in men by DDAVP not only reduced urine volume but that AVP causes an increase in the capacity of epithelial sodium channel (ENaC) receptors in the distal nephron to absorb sodium. This effect is solute-specific as the excretion of potassium and creatinine did not change. The retention of sodium or impaired sodium excretion can cause hypertension. Thus, hypertension in black individuals is thought to be salt-sensitive as an increase in salt intake will result in retention of sodium. AVP may also play a significant role in salt-sensitive hypertension in chronic kidney disease (CKD) patients due to a higher level of AVP causing activation of preglomerular V1a receptors and V2 receptors in collecting tubule (29).

Further it has been demonstrated that AVP, at levels much higher than needed to maintain an antidiuretic response, can cause smooth muscle contraction by acting on V1a receptors. However, this response is rapidly buffered by an intact autonomic nervous system and intact cardiovascular reflexes causing activation of the vagus nerve and bradycardia. It has been demonstrated in dogs that if the autonomic nervous system is disrupted, acute infusion of AVP will significantly elevate the mean arterial pressure (MAP). Mohring et al. studied the effect of AVP on BP in normal subjects and patients with idiopathic orthostatic hypotension presumably secondary to autonomic dysfunction. The results revealed that to increase MAP by 10 mm Hg, a 1000 times higher concentration of AVP was needed in normal subjects than in those with automatic insufficiency. These data suggest that a higher level of AVP in primary hypertension in humans can have significant impact on the cardiovascular system if the autonomic nervous system is disrupted (30).

In summary, the complex interrelationships of AVP, sodium, water, and other hormones in relation to the control of BP are of great interest and should continue to be the object of study.

## Chronic Kidney Disease Progression

Traditional risk factors for CKD progression such as diabetes, hypertension, and albuminuria are well described. However, numerous researchers have suggested that increased AVP levels can cause worsening CKD. We know that water intake and vasopressin levels are inversely related. Low 24 h urine volume and low water intake has been correlated with worsening CKD and is known to be associated with high AVP levels. Elevated urinary osmolality is not only associated with worsening CKD but also associated with initiation of dialysis after 72 months (31). In humans, the retrospective MDRD trial has confirmed this association and this has been supported by experimental studies in rats. In these studies, continuous infusion of DDAVP caused worsening albuminuria and CKD. On the contrary, when V2 receptors were selectively blocked, the outcome was opposite. Further, elevated copeptin levels, a surrogate marker for AVP, are associated with albuminuria and decreasing glomerular filtration rate (GFR) in kidney transplant patients (31).

Such observations support the hypothesis that V2 receptor stimulation is associated with worsening CKD. Alcohol and tobacco exert opposite influences on GFR. Alcohol blocks the V2 receptor and tobacco stimulates the V2 receptor. Thus, alcohol can decrease the fall in GFR and tobacco increase the fall in GFR (32, 33).

Arginine vasopressin may also cause worsening CKD by stimulating the renin angiotensin system. AVP interacts with V2 receptors causing direct stimulation of renin and indirectly it increases renin activity by reducing sodium load to the macula densa. In fact, DDAVP induced albuminuria and was associated with an increase in renin activity and this action was blocked with an ACE inhibitor. Increased RAAS activity causes constriction of efferent arteriole which causes hyperfiltration and glomerular damage with worsening proteinuria (34).

Arginine vasopressin is also known to affect GFR. In ADPKD, tolvaptan, a V2 receptor blocker, caused a significant slowing of reduction of GFR. Anderson et al. in his human experiments infused AVP in healthy volunteers for 2 h and he noted that there was a significant increase in creatinine clearance which was a reflection of higher GFR along with increased urine concentration and a decrease in fractional excretion of sodium and urea (2). Hyperfiltration may induce glomerulosclerosis, proteinuria, and worsening kidney disease. This effect may be more relevant with high protein intake, male gender, and black race (34).

In another study, a comparison was done between diabetic rats that have normal vasopressin secretion and rats with no vasopressin secretion. DM was generated by causing destruction of islets of Langerhans cells by infusing streptozotocin. Over time, rats that had diabetes and no vasopressin secretion had none of the signs of early diabetic nephropathy compared to rats with diabetes with intact vasopressin secretion. Also, urinary albumin excretion (UAE) occurred to a much lesser extent in diabetic vasopressin-deficient rats (35). Bardoux et al. has shown that in diabetic rats, if tolvaptan is used and urine osmolality is reduced close to serum osmolality, the rise of UAE was stopped (36). Desmopressin when infused in healthy people not only increased urine osmolality but also significantly increased UAE. In that study, urine creatinine did not change (37).

The above data have been cited to support a role for AVP in the progression of CKD.

## AVP, Klotho, and Aging

Arginine vasopressin may relate to the aging process through interactions with the transmembrane protein klotho. Klotho is widely expressed but it mainly influences the kidney and CNS. It also has an extracellular domain and can be released into the blood and the cerebrospinal fluid (CSF) and thus can also act as a hormone. Klotho is a major determinant of life-span (38). Klotho circulates in the blood and binds to receptors in various cells and increases cellular resistance to oxidative stress thus extending life-span. Binding of klotho to its receptors also suppress the intracellular insulin cascade also leading to increased resistance to oxidative stress which contributes to the anti-aging properties of klotho. Klotho-deficient rats have a decreased life-span, premature aging, and death occurs in less than 5 months. Apart from these effects, klotho deficiency can also cause muscular dystrophy, osteopenia, vascular calcification, and hearing loss which are other accelerated age-related disorders (38).

Klotho inhibits the 1-alpha hydroxylase enzyme in the kidney thus reducing the level of 1, 25 dihydroxycholecalciferol (1,25D) in the blood. Klotho deficiency causes increased production of 1,25D leading to hyperphosphatemia which in turn can increase vascular calcification. Thus, the reduced life-span in klotho deficiency, at least in part, can be explained by an increase in the 1,25D level. In klotho-deficient mice, life span was significantly increased by a low vitamin D diet (39).

Tang et al. studied the association between the klotho protein and dehydration. He conducted his study in hydrated and 36 h dehydrated mice. The study was also conducted in human embryonic kidney cells. Klotho transcript level was measured by polymerase chain reaction and protein abundance was detected by western blot analysis. He found that klotho transcript and abundance was significantly reduced in dehydrated mice. Dehydration causes an increase in serum osmolarity, plasma AVP, aldosterone, and 1,25D. Tang et al. exposed human embryonic kidney cells to a high level of AVP and aldosterone and he noted that the expression of klotho was significantly diminished by AVP and aldosterone; thus AVP may have a role in reducing klotho expression. Elderly diabetic patients are more prone to dehydration and may be at increased risk for renal injury, accelerated aging and early death possibly in association with secondary elevation of AVP levels and their effect on klotho (40).

## AVP and Social Behavior

Vasopressin and oxytocin receptors are widely distributed in the brain. V1a and V1b receptors are found in hippocampus, hypothalamus, and cerebral cortex. V1b receptors are also found on the pituitary and thus AVP may play a significant role in the regulation of the hypothalamic–pituitary–adrenal axis (HPA) in response to psychological stress. Oxytocin and AVP have a significant effect on psychological and cognitive functions. Endogenous AVP increases anxiety while endogenous oxytocin is anxiolytic. Thus, both neuropeptides have different psychological responses upon stimulation of their receptors and the balance of their actions determines social and emotional responses. Dysregulated balance between the actions of these peptides has been linked to various mental disorders such as depression, anxiety, schizophrenia, autism, personality disorders, and obsessive-compulsive disorders (OCDs). In patients suffering with depression, both plasma levels of AVP and levels of AVP in the paraventricular nucleus are significantly increased. During anxiety and OCD, plasma and CSF levels of AVP are significantly increased. Injection of hypertonic saline has induced panic symptoms in patients susceptible to panic attacks suggesting AVP may play a role in the induction of panic symptoms (41).

During acute schizophrenia, the plasma level of AVP is elevated. It has been suggested that the HPA response to psychological stress is abnormal in patients with schizophrenia but normal in response to physical stress. For example, CRH activates the HPA axis during physical stress whereas AVP may activate the HPA axis during psychological stress. This suggests that AVP may play a role in pathophysiology of schizophrenia. This hypothesis is supported by the actions of neuroleptic drugs like haloperidol which reduce plasma AVP levels along with the control of psychiatric symptoms. In contrast, phencyclidine (PCP) can induce severe symptoms of schizophrenia by elevating plasma AVP levels and by altering cerebral AVP receptor expression and distribution (42).

In autism, communication and reciprocal social interaction is impaired. It has been shown in humans that intranasal AVP affects social interaction. Further, autism has been linked to a mutation in V1a receptor genes raising the possibility that AVP may play a role in the impaired social behavior in autism (42).

Finally, intracerebral injection of AVP in animals has been linked to aggressive behavior and increased anxiety. Over expression of V1a receptors is linked to increased anxiety. Mutant mice lacking V1a receptors were noted to have reduced anxiety and impaired social recognition. Also, aggressive behavior in hamsters was blocked with an oral V1A receptor antagonist. Microinjection of a V1a receptor antagonist reduces anxiety in rats. Mouse null in V1a and V1b receptors had impaired social interactions but had normal motor functions which again suggests that AVP predominantly affects psychological functions. V1a null mice were also demonstrated to have reduced memory. In male rodents, AVP has been linked to partner preference and paternal care after mating and AVP antagonists altered this behavior (43).

## AVP and Cognitive Functions

Since AVP may have the above actions on behavior, it has been suggested that AVP may also have an effect on cognitive function. Cognitive functions may be influenced by hydration status and dehydration can increase AVP levels which may relate to changes in mentation.

That severe dehydration can cause major organ dysfunction and death is well known, but even mild dehydration and a loss of 1–2% of body weight can cause disturbances in cognitive function. Elderly diabetic patients and children are more prone to dehydration and thus more likely to have behavioral issues due to dehydration. Mild dehydration can occur during day-to-day activity. Ganio et al. studied the impact of dehydration on cognitive function and mood in an experiment conducted in 26 young males. He noted that mild dehydration was associated with deterioration in cognitive performance, visual vigilance, and an increase in tension, anxiety, and fatigue. People who were hyperhydrated had better memory functions than controls (44).

Spigt et al. studied the effect of dehydration on headache. He conducted an experiment in migraine patients and randomized them to receive placebo and 1.5 L of water daily. He noted that there was not only reduction in intensity but also the reduction in number of hours the patient suffered from headache in the hydrated patients (45).

Although none of these experiments directly linked AVP to changes in mood and cognitive function during dehydration, serum osmolality increases even in mild dehydration which results in an increase in AVP levels. It is therefore worthy of consideration that elevated AVP with its many cerebral actions and effects on behavior may also be related to the adverse cognitive effects of dehydration (46).

## AVP and Cellular Proliferation

Arginine vasopressin has been demonstrated to have an important role in cell proliferation and growth by directly stimulating protein synthesis and the production of growth factors which in turn cause cellular proliferation. AVP stimulates V1a receptors which stimulate the intracellular enzyme cascade leading to cell cycle progression and protein synthesis. The ability of AVP to induce cellular proliferation and protein synthesis has been studied by using radioactive thymidine. There was an increased uptake of radioactive thymidine when cells were exposed to AVP. This increased uptake was blocked by a V1a receptor antagonist but not by a V2 receptor antagonist (47).

Chiu et al. demonstrated the proliferative effect of AVP on rat intestinal epithelial cells. Wear and tear of small intestinal mucosa can cause a denuded area leading to cell migration from the margin and increased cellular proliferation causing restitution of the intestinal epithelium. Chiu et al. exposed intestinal epithelial cells to AVP without any other growth factors and the authors noted both migration and cellular proliferation of these cells in dose-dependent manner. This highlights the role of AVP in the maintenance of small intestinal epithelium. AVP is present in the crypt cells of the stomach and small intestine. Thus, AVP through its paracrine effect is involved in the repair of tissues (48).

Mesangial cells play a vital role in the microcirculation of the glomerulus. Proliferation of glomerular cells can cause occlusion of the capillaries. Many factors may cause proliferation of the mesangial cells such as AVP, angiotensin-2, and endothelin-1, which are present systemically and also produced locally. AVP is known to cause proliferation of glomerular mesangial cells and vascular smooth muscle cells by stimulating V1a receptors. In fact, it has been shown that antagonists to V1a receptors given on a chronic basis can inhibit mesangial cell proliferation (49). Another mechanism by which AVP induces mesangial cellular proliferation is by inducing autocrine secretion of vascular endothelial growth factor (VEGF). AVP acts on V1a receptor which in turn causes secretion of VEGF from mesangial cells. VEGF is a potent mitogen which in turn acts in an autocrine manner to cause mesangial cell hypertrophy and proliferation. AVP also causes stimulation of proliferation of fibroblasts present in the mesangium which cause increased deposition of type 4 collagen leading to impairment of glomerular microcirculation and injury (50).

Mackinnon et al. has shown that tumor cells of small cell lung cancer (SCLC) demonstrate V1a receptors. SCLC cells secrete neuropeptides and also have receptors for neuropeptides. In a clinical trial conducted by Sethi et al., CD133 cancer cells from SCLC patients resistant to the chemotherapeutic agent etoposide were treated with a V1a receptor blocker called peptide 1 and it was noted that these CD133 cancer cells which were resistant to etoposide now became sensitive (51).

Arginine vasopressin also plays a major role in organ regeneration. In an animal experiment, partial hepatectomy was done in rat liver. In this study, the plasma AVP level increased significantly almost immediately and it peaked in 1 h by activation of the hypothalamic nuclei. There was complete restoration of liver mass in a few days suggesting that AVP has a significant role in liver regeneration. In another experiment, partial hepatectomy was also done in rats. The action of AVP was blocked by using a V1a receptor antagonist. V1a antagonism caused significantly decreased DNA synthesis and decreased liver mass restoration in the first 24–48 h. The significance of AVP in liver regeneration was also documented in rats of the Brattleboro strain. These rats have hereditary AVP deficiency. Partial hepatectomy was done in these rats and liver generation was impaired and when AVP was administered liver regeneration increased. This further demonstrates the significance of AVP in hepatocyte regeneration (3).

The effect of AVP on protein synthesis was also demonstrated in the neonatal mouse cardiomyocyte. Neonatal mouse cardiomyocytes exposed to AVP for 24 h had increased protein synthesis and an increase in the cell surface area in a dose-dependent manner. This effect was lost in the presence of a V1a antagonist (3).

The data summarized above suggest an important role for AVP and AVP-blocking drugs in regard to cellular proliferation, regeneration, and the growth of neoplasms.

## AVP and Inflammation

Various inflammatory states such as pneumonia, encephalitis, malaria, and adult respiratory distress syndrome are associated with the development of inflammatory cytokines such as interleukin 1 (IL-1) and interleukin 6 (IL-6) which have been shown to be associated with the release of AVP (52). It has also been shown that injection of IL-6 antibodies in the brain blocked the activation of AVP-secreting neurons in response to injected lipopolysaccharide (LPS). In humans, a temporal relationship was found between injection of IL-6 and elevation of serum AVP levels. Serum AVP levels were elevated within 2 h of IL-6 injection. This suggests that IL-6 stimulates AVP-secreting neurons, increasing the serum levels of AVP which may lead to the development of hyponatremia. The mechanism of the release of AVP in response to the peripheral injection of cytokines such as IL-6 is unclear. However, it is suggested that IL-6 can diffuse across the BBB and it is also actively secreted inside the brain BBB pericytes. Bacteremia in pediatric patients is associated with an early rise of CRP levels. It has been shown that elevated CRP is associated with hyponatremia. Thus, hyponatremia could be used as a marker of inflammation in certain settings (53).

Further, not only does the inflammatory response mediated by cytokines stimulate the release of AVP, but it has been shown that AVP has immunomodulatory actions. Boyd et al. in his animal experiments has shown that AVP can modulate the immune response by downregulating innate immunity when exposed to an antigen (54). LPS is a potent endotoxin and can stimulate an innate immune response when given systemically. IL-6 is a marker of innate immune response. Boyd et al. in his experiments with rats, injected intraperitoneal LPS. LPS dramatically increased the level of IL-6 in the serum and the lung compared to controls that were given intraperitoneal saline. The IL-6 level was monitored in the serum and the lung after low-dose AVP injection. The investigators noted a significant reduction of IL-6 in the lung after AVP exposure but no reduction of IL-6 in the serum. This shows that the immunomodulatory action of AVP was lung-specific. They also noted that the immunomodulatory action of AVP was significantly suppressed when mice were pretreated with a V2 receptor antagonist. This experiment demonstrates that AVP reduces inflammation probably through V2 receptors in the lungs. They also confirmed the immunomodulatory response of AVP in human alveolar epithelial cells. Low-dose AVP injected in mice did not affect hemodynamics but significantly reduced the innate immune response. Thus, the efficacy of AVP in septic shock may be due to both its vasoactive and immunomodulatory properties (54).

Corticotropin-releasing hormone and AVP are not only present in the cells within the hypothalamus but they are localized in the cells of the immune system. AVP has been found in rat B lymphocytes and thymic epithelial cells. AVP has also been found in human peripheral blood mononuclear cells (PBMC). Human PBMC have AVP receptors. Thus, in response to inflammation, AVP can stimulate the production of cytokines and antibodies through these receptors. AVP and CRH are released not only from the hypothalamus but also from peripheral immune cells in response to stress and inflammation (52). In rats, the expression of AVP is increased in B cells during arthritis. In resting PBMC from healthy humans, the concentration of CRH and AVP is low but it is significantly increased in response to inflammation. In addition, it has been shown in mice that blocking of V1a receptors in mice altered the humoral immunity.

The HPA axis plays a significant role during inflammation. Cytokines released during inflammation stimulate the release of CRH and AVP from the hypothalamus. AVP and CRH act synergistically on the pituitary to release ACTH. ACTH stimulates the adrenal cortex to release corticosteroid which in turn suppresses inflammation. Defective response of the hypothalamus to inflammation leads to chronic inflammatory states due to diminished secretion of ACTH and corticosteroid. Lewis rats have a defective neuroendocrine response to inflammation and this may contribute to their chronic inflammatory state. HPA axis response is diminished in patients with rheumatoid arthritis which is manifested by a low cortisol level for the degree of joint inflammation. AVP is also a pro-inflammatory peptide which can stimulate the release of prolactin, a pro-inflammatory peptide, which can also exacerbate inflammation. AVP stimulates the release of cytokines, increases T helper 1 cell actions, and augments mixed lymphocytic response further worsening inflammation. Immunoneutralization of serum AVP in rats has been shown to diminish inflammation. Thus, excessive production of AVP due to an abnormal HPA axis may contribute to the chronic inflammatory state (55).

## AVP and Infections

Hormones including AVP play an important role in the manner in which responses to infection are modulated. The upper and lower urinary tract is generally considered a sterile environment and usually is not colonized by bacteria. Urinary tract infections are often caused by uropathogenic *Escherichia coli* (UPEC) by retrograde ascent of bacteria from the urinary bladder. UPEC has special affinity to urinary bladder epithelium and renal tubular epithelial cells (RTEC). Pyelonephritis can cause renal insufficiency in children and adults and also can occur after renal transplantation.

The initial response of the body to any infection is in the form of innate immunity. Innate immunity is considered as the first line of defense and defends the host from infections in a non-specific manner. Toll like receptors (TLRs) are pattern recognition receptors. They identify pathogen-associated molecular pattern (PAMP) resulting in activation of the innate immune response. TLRs have a transmembrane domain and are present widely. They are present on hematopoietic cells and different epithelial cell types like intestinal cells and the RTEC of the collecting duct. RTEC play a significant role in activating the innate immune response and are the initial defense following the colonization of the kidney by UPEC.

Lipopolysaccharide is an essential component of the cell wall of Gram negative rods (GNRs). TLR4 present on the apical RTEC recognizes the LPS and in turn activates a cascade of enzymatic reactions which result in activation of nuclear transcription factors (NF). NF in turn stimulates DNA to produce pro-inflammatory cytokines. These cytokines activate the cells involved in innate immunity thus causing destruction of the GNR. Thus, TLRs are essential in activating innate immunity, mounting host defense, and preventing pyelonephritis (56).

Arginine vasopressin plays an important role in the reabsorption of water by increasing cyclic AMP levels. High cyclic AMP levels cause reduction of the expression of the adhesion and signaling molecules in the collecting duct cells after bacterial translocation making innate immunity ineffective and impairing renal bacterial clearance. DDAVP has been shown to inhibit LPS-induced activation of NF thus inhibiting the release of pro-inflammatory cytokines. V2 receptor antagonists prevent the effects of DDAVP on inflammation thus causing increased secretion of pro-inflammatory mediators and recruitment of neutrophils. This shows that AVP can play a role in interfering with TR4-mediated signaling, downregulating intrarenal inflammation and play a significant role in influencing innate immunity. Children with pyelonephritis can exhibit high AVP levels. Experiments in rats have shown that water deprivation significantly increases the risk of pyelonephritis and sustained water diuresis can cure enterococcal-induced pyelonephritis. Elderly patients are prone to dehydration and this in part may explain the high incidence of pyelonephritis in elderly patients. Hyperhydration decreases AVP levels and thus may reinforce innate immunity, preventing and limiting pyelonephritis. Thus, hormonal control of innate immunity may to some degree explain susceptibility to pyelonephritis in various individuals (57).

## AVP and Hemostasis

Desmopressin, a synthetic derivative of vasopressin, was initially used for the treatment of diabetes insipidus. Later, it was demonstrated that DDAVP can also be used for the treatment of von Willebrand’s disease and mild hemophilia as a transfusion saving agent during major surgeries characterized by large blood loss. It is also used in the treatment of bleeding due to platelet dysfunction from the use of aspirin, ticlopidine, uremia, or chronic liver disease.

Desmopressin exerts its hemostatic effect by increasing the serum levels of von Willebrand factor (vWF), factor VIII, and tissue plasminogen activator (t-PA). vWF is a glycoprotein produced by endothelial cells, with a small contribution by platelets. It exerts its hemostatic effect by binding platelets to the endothelial cell and also by being a carrier protein for factor VIII reducing its renal clearance and enzymatic degradation. t-PA and vWF are produced by endothelial cells and are stored in secretory granules called Weibel-palade (WP) bodies. DDAVP acts on endothelial cells and stimulates the release of t-PA and vWF increasing serum levels. The response to injected DDAVP is seen within 30 min which suggests that the increase in the level of vWF and t-PA is not due to increased production but is due to increased release from WP bodies. This explains the tachyphylaxis to repeat doses of DDAVP given at short intervals. It is suggested that factor VIII is produced by endothelial cells and is localized in the WP bodies along with vWF and t-PA and is released from the WP bodies upon stimulation by DDAVP. The response to DDAVP is variable among patients, thus a therapeutic trial should be given to estimate individual responses prior to therapeutic use. The response to intranasal DDAVP is much less and slower compared to DDAVP given by the subcutaneous (SC) route or intravenously (IV). Thus, during an emergency, the SC or IV route is preferred (58).

Desmopressin exerts its action through V2 receptors which are present widely on the endothelial cell. DDAVP exerts its action even in patients who have undergone bilateral nephrectomy suggesting that extrarenal V2 receptors are involved in the release of vWF. Further, a selective V2 receptor antagonist blocked the response to DDAVP on endothelial cells. DDAVP also failed to produce a response in patients with X-linked diabetes insipidus in which there is a mutation of renal and extrarenal V2 receptors. DDAVP causes an increase in cyclic AMP in the endothelial cells which stimulates an enzymatic cascade releasing the factors from the WP bodies. Further, DDAVP is unable to exert its action on human umbilical vein epithelial cells (HUVECs) which lack V2 receptors confirming that DDAVP acts on endothelial cells through V2 receptors (59).

## AVP and the Hypothalamic–Pituitary–Adrenal Axis

The HPA axis plays a vital role in the maintenance of homeostasis in response to stress. The HPA axis reacts to stress by releasing ACTH which acts on the adrenal cortex to release corticosteroids. Corticosteroid influences multiple physiological functions in response to stress. Dysfunction of the HPA axis can result in a chronic inflammatory state due to the deficiency of corticosteroids. CRH mainly influences the release of ACTH, however, AVP also plays a significant role in releasing ACTH in response to stress by directly acting on V1b receptors in the pituitary and AVP also acts synergistically by potentiating the actions of CRH. Intraperitoneal or oral administration of a V1b receptor antagonist prevents the increase in the ACTH level in response to injected AVP. Moreover, in mice lacking V1b receptors, the ACTH level was not increased in response to injected AVP. In mice, the increase in the ACTH level was also prevented by a V1b receptor blocker in response to stress such as acute restraint and LPS injection. This demonstrates that CRH can not compensate for the V1b receptor blockade suggesting an important role of AVP in increasing ACTH levels in response to acute stress. It has also been suggested that AVP does not play an important role in the resting hormone level of ACTH and corticosteroid. This was shown in Brattleboro rats which congenitally lack AVP. In these rats, basal level of ACTH and corticosteroid did not differ compared to controls. However, the stress-induced ACTH level was markedly impaired in these Brattleboro rats compared to controls. In has also been stated that in response to chronic stress such as repeated restraints, ACTH level is increased mainly by AVP rather than CRH (3).

Apart from its action of releasing ACTH, AVP plays a significant role in regulating the release of the hormones of the adrenal gland by acting on V1a and V1b receptors. V1a receptors are located in the adrenal cortex and V1b receptors are located on the chromaffin cells in the adrenal medulla. AVP is not only produced centrally but it is also produced locally in many tissues and this has been demonstrated in the adrenal medulla by the presence of the immunoreactivity of AVP along with epinephrine and norepinephrine in the chromaffin cells in the adrenal medulla. It has been suggested that AVP is produced and secreted by chromaffin cells in response to acetylcholine and stress. AVP acts on V1b receptors on the chromaffin cells secreting epinephrine and norepinephrine in response to acute and chronic stress acting in an autocrine and paracrine manner. The significance of V1b receptors was demonstrated in mice lacking V1b receptors. Basal levels of epinephrine and norepinephrine were similar in V1b-deficient mice compared to controls. However, in response to acute and chronic stress, the levels of epinephrine and norepinephrine were significantly less (60).

Arginine vasopressin also acts on V1a receptors in the adrenal cortex and stimulates hypertrophy and hyperplasia of the cells in the adrenal cortex thus resulting in the enlargement of the zona glomerulosa. This results in increased production and secretion of aldosterone and corticosteroid. This response was blocked by a V1a receptor antagonist and in mice which lack V1a receptors. Brattleboro rats, which are deficient in vasopressin, have diminished secretion of aldosterone suggesting that AVP stimulates aldosterone secretion directly independent of its action on ACTH secretion (61). It has also been suggested that AVP may play an important role in the pathophysiology of Cushing’s syndrome. Cushing’s syndrome can be caused by an adrenal tumor or hyperplasia which is ACTH-independent. It has been demonstrated that these tumor cells have either over expressed V1a receptors or have a mutated form of V1a receptor leading to increased sensitivity to AVP causing increased secretion of corticosteroid (62).

Thus, AVP plays an important role in regulating the HPA by centrally releasing AVP and also the peripheral production of AVP influences secretion of hormones of the adrenal gland by its autocrine and paracrine functions.

## Conclusion

The above comprehensive review describes the various nonpressor and nonantidiuretic actions of AVP. Appreciation of these relationships should stimulate further research activities in these areas of great potential importance.

## Conflict of Interest Statement

The authors declare that the research was conducted in the absence of any commercial or financial relationships that could be construed as a potential conflict of interest.
